# Rupatadine

**DOI:** 10.1107/S1600536813014256

**Published:** 2013-05-31

**Authors:** Manpreet Kaur, Jerry P. Jasinski, Zane A. Luopa, Neeraj Kumar, Nilesh G. Patel, Omprakash Gudaparthi, H. S. Yathirajan

**Affiliations:** a Department of Studies in Chemistry, University of Mysore, Manasagangotri, Mysore 570 006, India; b Department of Chemistry, Keene State College, 229 Main Street, Keene, NH 03435-2001, USA; c CR & D, Cadila Pharmaceuticals Ltd, 1389, Trasad Road, Dholka, Ahmedabad 387 810, Gujarat, India

## Abstract

In the title compound (systematic name: 8-chloro-11-{1-[(5-methyl­pyridin-3-yl)meth­yl]piperidin-4-yl­idene}-6,11-di­hydro-5*H*-benzo[5,6]cyclo­hepta­[1,2-*b*]pyridine), C_26_H_26_ClN_3_, the dihedral angle between the mean planes of the chloro­phenyl and cyclo­hepta­[1,2-*b*]pyridinyl rings fused to the cyclo­heptane ring is 56.6 (1)°. The mean planes of the cyclo­hepta­[1,2-*b*]pyridinyl and 5-methyl­pyridin-3-yl rings are twisted by 64.9 (4)°. The central piperizene group is in a slightly distorted chair configuration. A weak intra­molecular C—H⋯N inter­action is observed between the cyclo­hepta­[1,2-*b*]pyridinyl and piperidin-4-yl­idene moieties.

## Related literature

For the pharmacological importance of rupatadine, see: Kean & Plosker (2007 ▶); Merlos *et al.* (1997 ▶); Mullol *et al.* (2008 ▶); Picado (2006 ▶). For the reported synthesis methodology of rupatadine, see: Agarwal *et al.* (2008 ▶). For standard bond lengths, see: Allen *et al.* (1987 ▶).
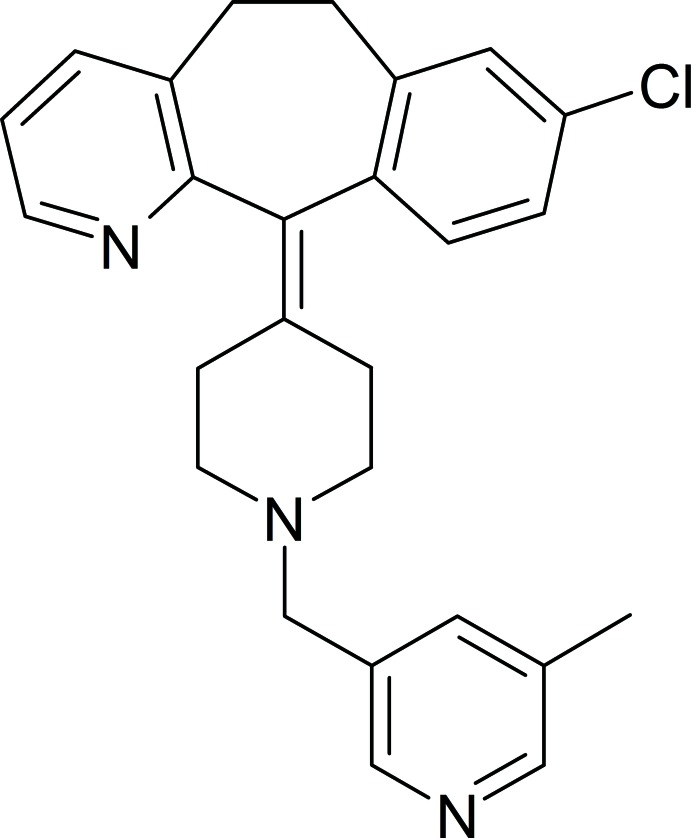


## Experimental

### 

#### Crystal data

C_26_H_26_ClN_3_
*M* *_r_* = 415.95
Monoclinic, 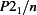

*a* = 10.2655 (3) Å
*b* = 11.3341 (4) Å
*c* = 18.8111 (6) Å
β = 90.874 (3)°
*V* = 2188.43 (11) Å^3^
*Z* = 4
Cu *K*α radiation
μ = 1.67 mm^−1^
*T* = 173 K
0.42 × 0.38 × 0.22 mm

#### Data collection

Agilent Xcalibur (Eos, Gemini) diffractometer
Absorption correction: multi-scan (*CrysAlis PRO* and *CrysAlis RED*; Agilent, 2012 ▶) *T*_min_ = 0.673, *T*_max_ = 1.000
13849 measured reflections
4281 independent reflections
3565 reflections with *I* > 2σ(*I*)
*R*_int_ = 0.026

#### Refinement

*R*[*F* ^2^ > 2σ(*F* ^2^)] = 0.041
*wR*(*F* ^2^) = 0.122
*S* = 1.05
4281 reflections
273 parameters
H-atom parameters constrained
Δρ_max_ = 0.22 e Å^−3^
Δρ_min_ = −0.29 e Å^−3^

### 

Data collection: *CrysAlis PRO* (Agilent, 2012 ▶); cell refinement: *CrysAlis PRO*; data reduction: *CrysAlis RED* (Agilent, 2012 ▶); program(s) used to solve structure: *SHELXS97* (Sheldrick, 2008 ▶); program(s) used to refine structure: *SHELXL2012* (Sheldrick, 2008 ▶); molecular graphics: *OLEX2* (Dolomanov *et al.*, 2009 ▶); software used to prepare material for publication: *OLEX2*.

## Supplementary Material

Crystal structure: contains datablock(s) global, I. DOI: 10.1107/S1600536813014256/hg5317sup1.cif

Structure factors: contains datablock(s) I. DOI: 10.1107/S1600536813014256/hg5317Isup2.hkl

Supplementary material file. DOI: 10.1107/S1600536813014256/hg5317Isup3.cml

Additional supplementary materials:  crystallographic information; 3D view; checkCIF report

## Figures and Tables

**Table 1:** Hydrogen-bond geometry (Å, °)

*D*—H⋯*A*	*D*—H	H⋯*A*	*D*⋯*A*	*D*—H⋯*A*
C19—H19*B*⋯N1	0.99	2.60	3.229 (2)	121

**Fig. 1.**
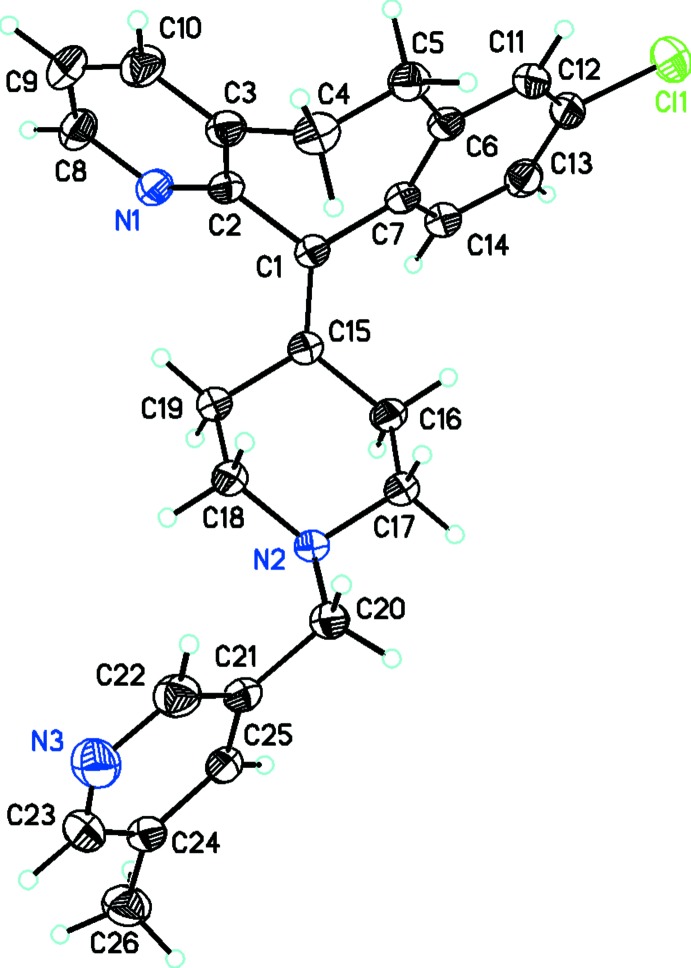
Molecular structure of the title compound showing the atom labeling scheme and 30% probability displacement ellipsoids.

**Fig. 2.**
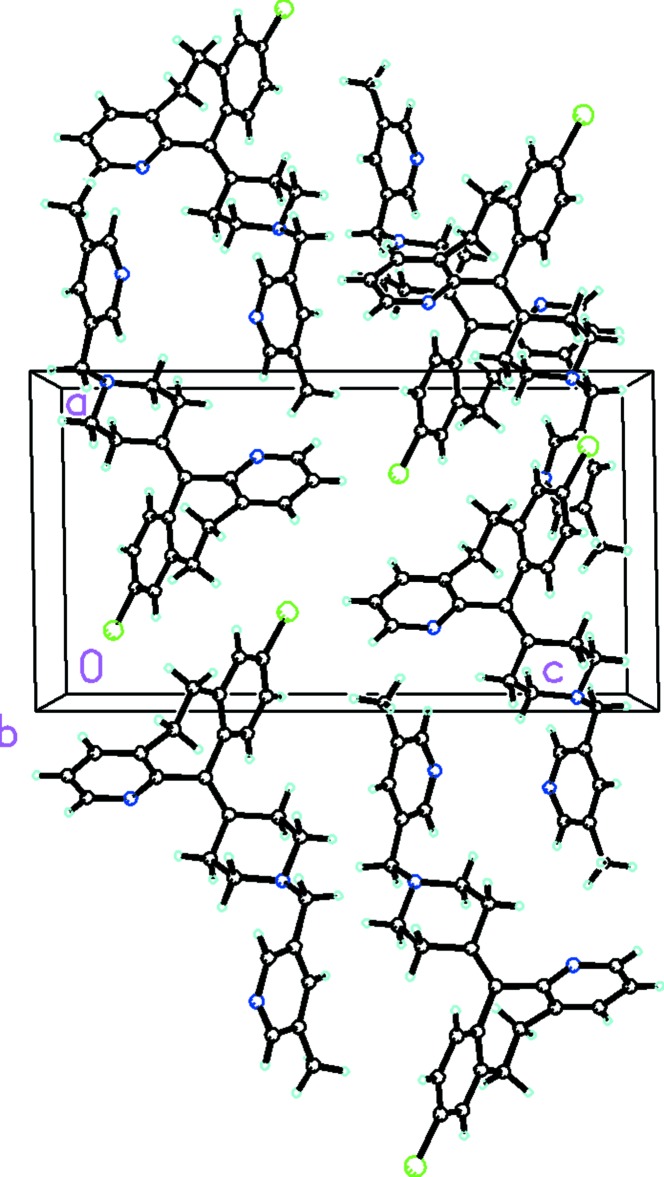
Packing diagram of the title compound viewed along the b axis.

**Fig. 3.**
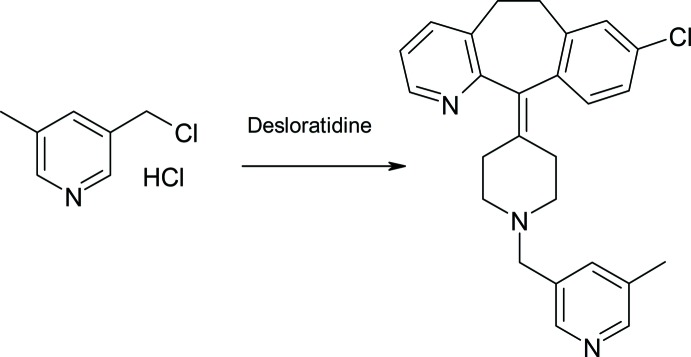
Reaction scheme for the synthesis of rupatadine free base.

## supplementary crystallographic information

### Comment

Rupatadine (IUPAC Name: 8-Chloro-6,11-dihydro-11-[1-[(5-methyl-3- pyridinyl) methyl]-4-piperidinylidene]-5H-benzo[5,6]cyclohepta[1,2-b] pyridine) is a non-sedating antihistamine showing a rapid onset of action and a good safety profile even in prolonged treatment periods of a year (Picado, 2006; Mullol *et al.*, 2008). A review of its use in the management of allergic disorders is published (Kean & Plosker, 2007). Rupatadine has shown as inhibition deregulation, induced by the immunological and non-immunological stimulants and the inhibition of release of cytokines, particularly the tumor necrosis factor alpha (TNF-alpha) in human mastocytes and monocytes (Picado, 2006). In vitro metabolism studies indicate that rupatadine is metabolized mainly by the cytochrome P-450 in liver (Merlos *et al.*, 1997). In view of the importance of the title compound, (I), C_26_H_26_ClN_3_, we have synthesized rupatadine free base based on a reported method (Agarwal *et al.*, 2008) and its single crystal structure is reported herin.

In (I), the dihedral angle between the mean planes of the chlorophenyl and cyclohepta[1,2-b]pyridinyl rings fused to the cycloheptane ring is 56.6 (1)° (Fig. 1). The mean planes of the cyclohepta[1,2-b]pyridinyl and 5-methyl-3-pyridinyl rings are twisted by 64.9 (4)°. The central 6-membered piperizene group adopts a slightly distorted chair configuration with puckering parameters Q, θ and φ of 0.5613 (16)Å, 3.31 (16)°, and 348 (3)°, respectively. A weak C—H···O intramolecular interaction is observed between the cyclohepta[1,2-b]pyridinyl and 4-piperidinylidene moieties. In the crystal, the molecules pack in a normal head-to tail dimer-like arrangement (Fig. 2).

### Experimental

4-methyl-3-chloromethyl pyridine hydrochloride (3.5 g, 0.02 mol), desloratadine (6.2 g, 0.02 mol), potassium carbonate (6.9 g, 0.05 mol) was charged into acetonitrile (30 ml) Fig. 3). The reaction mass was heated to 313–318 K and stirred for 10-12 h (Agarwal *et al.*, 2008). The reaction mass was cooled to 298–303 K and the inorganic material filtered. The solvent was removed under reduced pressure. Toluene (40 ml) was added to residue and heated to 328-333 K to get a clear solution. The toluene layer was washed with a saturated sodium chloride solution (40 ml) and water (25 ml). Half the quantity of toluene was distilled out under vacuum and single crystals were grown from toluene using the slow evaporation technique (m. p.: 409–410 K).

### Refinement

All of the H atoms were placed in their calculated positions and then refined using the riding model with Atom—H lengths of 0.95Å (CH), 0.99Å (CH_2_) or 0.98Å (CH_3_). Idealised Me was refined as a rotating group: C26(H26A,H26B,H26C). Isotropic displacement parameters for these atoms were set to 1.2 (CH, CH_2_) or 1.5 (CH_3_ times *U*_eq_ of the parent atom.

### Crystal data

**Table:**

C_26_H_26_ClN_3_	*F*(000) = 880
*M**_r_* = 415.95	*D*_x_ = 1.262 Mg m^−^^3^
Monoclinic, *P*2_1_/*n*	Cu *K*α radiation, λ = 1.5418 Å
*a* = 10.2655 (3) Å	Cell parameters from 4864 reflections
*b* = 11.3341 (4) Å	θ = 3.9–72.2°
*c* = 18.8111 (6) Å	µ = 1.67 mm^−^^1^
β = 90.874 (3)°	*T* = 173 K
*V* = 2188.43 (11) Å^3^	Irregular, clear orangish orange
*Z* = 4	0.42 × 0.38 × 0.22 mm

### Data collection

**Table:**

Agilent Xcalibur (Eos, Gemini) diffractometer	4281 independent reflections
Radiation source: Enhance (Cu) X-ray Source	3565 reflections with *I* > 2σ(*I*)
Graphite monochromator	*R*_int_ = 0.026
Detector resolution: 16.0416 pixels mm^-1^	θ_max_ = 72.4°, θ_min_ = 4.6°
ω scans	*h* = −11→12
Absorption correction: multi-scan (*CrysAlis PRO* and *CrysAlis RED*; Agilent, 2012)	*k* = −8→13
*T*_min_ = 0.673, *T*_max_ = 1.000	*l* = −21→23
13849 measured reflections	

### Refinement

**Table:**

Refinement on *F*^2^	Hydrogen site location: inferred from neighbouring sites
Least-squares matrix: full	H-atom parameters constrained
*R*[*F*^2^ > 2σ(*F*^2^)] = 0.041	*w* = 1/[σ^2^(*F*_o_^2^) + (0.0616*P*)^2^ + 0.3876*P*] where *P* = (*F*_o_^2^ + 2*F*_c_^2^)/3
*wR*(*F*^2^) = 0.122	(Δ/σ)_max_ < 0.001
*S* = 1.05	Δρ_max_ = 0.22 e Å^−^^3^
4281 reflections	Δρ_min_ = −0.29 e Å^−^^3^
273 parameters	Extinction correction: *SHELXL2012* (Sheldrick, 2008), Fc^*^=kFc[1+0.001xFc^2^λ^3^/sin(2θ)]^-1/4^
0 restraints	Extinction coefficient: 0.0043 (3)
Primary atom site location: structure-invariant direct methods	

### Special details

**Table:**

Experimental. (HPLC purity 99.75 %) FT IR (KBr) : 1350.2, 1475.6, 1583.6; 1H NMR (300 MHz, DMSO d6) δ 2.072 (s, 1H), 2.127-2.164 (m, 3H), 2.247 (s, 3H), 2.264-2.320 (m, 2H), 2.545-2.580 (m, 2H), 2.725-2.827 (m, 2H), 3.217-3.324 (m, 2H), 3.406 (s, 2H), 7.011-7.038 (d, 1H), 7.124-7.193 (m, 2H), 7.242-7.248 (d, 1H), 7.474-7.537 (m, 2H), 8.250-8.313 (dd, 3H); MS m/z (EI): 416 (M + 1).
Geometry. All esds (except the esd in the dihedral angle between two l.s. planes) are estimated using the full covariance matrix. The cell esds are taken into account individually in the estimation of esds in distances, angles and torsion angles; correlations between esds in cell parameters are only used when they are defined by crystal symmetry. An approximate (isotropic) treatment of cell esds is used for estimating esds involving l.s. planes.

### Fractional atomic coordinates and isotropic or equivalent isotropic displacement parameters (Å^2^)

**Table:**

	*x*	*y*	*z*	*U*_iso_*/*U*_eq_	
Cl1	0.21448 (5)	0.23499 (6)	0.09566 (3)	0.0826 (2)	
N1	0.76596 (13)	0.40249 (13)	0.35326 (7)	0.0484 (3)	
N2	0.98891 (11)	0.58991 (11)	0.10639 (6)	0.0391 (3)	
N3	1.29611 (17)	0.88557 (15)	0.14524 (10)	0.0684 (5)	
C1	0.69993 (14)	0.44001 (13)	0.23053 (7)	0.0386 (3)	
C2	0.69596 (14)	0.46981 (14)	0.30801 (8)	0.0399 (3)	
C3	0.61966 (15)	0.56496 (14)	0.32955 (8)	0.0443 (4)	
C4	0.54486 (17)	0.63427 (15)	0.27422 (9)	0.0509 (4)	
H4A	0.6038	0.6550	0.2350	0.061*	
H4B	0.5137	0.7087	0.2956	0.061*	
C5	0.42840 (16)	0.56586 (15)	0.24409 (9)	0.0487 (4)	
H5A	0.3693	0.5488	0.2839	0.058*	
H5B	0.3808	0.6187	0.2108	0.058*	
C6	0.45366 (15)	0.45093 (14)	0.20564 (8)	0.0425 (3)	
C7	0.57478 (14)	0.39337 (14)	0.19947 (7)	0.0394 (3)	
C8	0.76056 (19)	0.43009 (18)	0.42245 (9)	0.0576 (5)	
H8	0.8101	0.3838	0.4552	0.069*	
C9	0.6876 (2)	0.52139 (19)	0.44884 (9)	0.0626 (5)	
H9	0.6863	0.5373	0.4984	0.075*	
C10	0.61619 (18)	0.58930 (16)	0.40146 (9)	0.0558 (4)	
H10	0.5647	0.6528	0.4183	0.067*	
C11	0.34414 (16)	0.39908 (17)	0.17349 (9)	0.0519 (4)	
H11	0.2620	0.4371	0.1767	0.062*	
C12	0.35305 (16)	0.29398 (17)	0.13731 (9)	0.0531 (4)	
C13	0.46934 (18)	0.23397 (16)	0.13272 (9)	0.0525 (4)	
H13	0.4744	0.1604	0.1087	0.063*	
C14	0.57883 (16)	0.28467 (15)	0.16437 (8)	0.0456 (4)	
H14	0.6596	0.2440	0.1621	0.055*	
C15	0.80824 (14)	0.45528 (14)	0.19264 (8)	0.0401 (3)	
C16	0.81702 (16)	0.43718 (15)	0.11334 (8)	0.0468 (4)	
H16A	0.7302	0.4154	0.0938	0.056*	
H16B	0.8778	0.3716	0.1035	0.056*	
C17	0.86446 (14)	0.54878 (15)	0.07714 (8)	0.0446 (4)	
H17A	0.8738	0.5333	0.0257	0.053*	
H17B	0.7984	0.6116	0.0826	0.053*	
C18	0.97594 (15)	0.61314 (15)	0.18277 (8)	0.0445 (4)	
H18A	0.9108	0.6764	0.1897	0.053*	
H18B	1.0604	0.6412	0.2024	0.053*	
C19	0.93417 (14)	0.50352 (15)	0.22298 (8)	0.0440 (4)	
H19A	1.0030	0.4426	0.2199	0.053*	
H19B	0.9227	0.5233	0.2738	0.053*	
C20	1.02633 (15)	0.69742 (15)	0.06862 (8)	0.0462 (4)	
H20A	0.9631	0.7606	0.0795	0.055*	
H20B	1.0215	0.6823	0.0168	0.055*	
C21	1.16119 (15)	0.73962 (13)	0.08791 (8)	0.0412 (3)	
C22	1.17979 (18)	0.84145 (16)	0.12707 (10)	0.0566 (4)	
H22	1.1046	0.8829	0.1421	0.068*	
C23	1.39933 (18)	0.82602 (18)	0.12289 (10)	0.0604 (5)	
H23	1.4831	0.8563	0.1350	0.072*	
C24	1.39372 (16)	0.72266 (16)	0.08313 (9)	0.0485 (4)	
C25	1.27098 (15)	0.67932 (15)	0.06625 (8)	0.0432 (3)	
H25	1.2621	0.6083	0.0398	0.052*	
C26	1.51532 (18)	0.6591 (2)	0.06151 (12)	0.0698 (6)	
H26A	1.5056	0.5745	0.0709	0.105*	
H26B	1.5899	0.6897	0.0889	0.105*	
H26C	1.5297	0.6715	0.0107	0.105*	

### Atomic displacement parameters (Å^2^)

**Table:**

	*U*^11^	*U*^22^	*U*^33^	*U*^12^	*U*^13^	*U*^23^
Cl1	0.0574 (3)	0.1179 (5)	0.0722 (3)	−0.0312 (3)	−0.0097 (2)	−0.0091 (3)
N1	0.0462 (7)	0.0604 (8)	0.0386 (7)	0.0055 (6)	0.0025 (5)	0.0016 (6)
N2	0.0342 (6)	0.0489 (7)	0.0344 (6)	−0.0019 (5)	0.0044 (5)	−0.0008 (5)
N3	0.0712 (11)	0.0625 (10)	0.0716 (11)	−0.0165 (8)	0.0050 (8)	−0.0175 (8)
C1	0.0391 (7)	0.0420 (8)	0.0349 (7)	−0.0004 (6)	0.0034 (6)	0.0015 (6)
C2	0.0383 (7)	0.0460 (8)	0.0356 (7)	−0.0052 (6)	0.0056 (6)	0.0020 (6)
C3	0.0457 (8)	0.0445 (8)	0.0430 (8)	−0.0044 (7)	0.0067 (6)	−0.0008 (7)
C4	0.0605 (10)	0.0402 (8)	0.0523 (9)	0.0026 (7)	0.0079 (7)	0.0035 (7)
C5	0.0476 (9)	0.0516 (9)	0.0469 (9)	0.0087 (7)	0.0051 (7)	0.0107 (7)
C6	0.0408 (8)	0.0505 (9)	0.0364 (7)	−0.0010 (6)	0.0049 (6)	0.0099 (6)
C7	0.0396 (7)	0.0459 (8)	0.0330 (7)	−0.0040 (6)	0.0050 (6)	0.0063 (6)
C8	0.0623 (11)	0.0736 (12)	0.0367 (8)	0.0052 (9)	−0.0012 (7)	0.0043 (8)
C9	0.0730 (12)	0.0789 (13)	0.0362 (8)	0.0016 (10)	0.0048 (8)	−0.0107 (8)
C10	0.0629 (11)	0.0555 (10)	0.0493 (9)	0.0025 (8)	0.0084 (8)	−0.0122 (8)
C11	0.0392 (8)	0.0706 (11)	0.0459 (9)	−0.0030 (7)	0.0033 (7)	0.0087 (8)
C12	0.0449 (9)	0.0717 (12)	0.0427 (8)	−0.0173 (8)	−0.0017 (7)	0.0056 (8)
C13	0.0598 (10)	0.0542 (10)	0.0435 (9)	−0.0123 (8)	0.0018 (7)	−0.0012 (7)
C14	0.0449 (8)	0.0495 (9)	0.0426 (8)	−0.0014 (7)	0.0037 (6)	0.0019 (7)
C15	0.0386 (7)	0.0457 (8)	0.0360 (7)	−0.0022 (6)	0.0034 (6)	−0.0008 (6)
C16	0.0451 (8)	0.0584 (10)	0.0370 (8)	−0.0105 (7)	0.0075 (6)	−0.0078 (7)
C17	0.0387 (8)	0.0608 (10)	0.0343 (7)	−0.0050 (7)	0.0030 (6)	−0.0020 (7)
C18	0.0418 (8)	0.0552 (9)	0.0364 (7)	−0.0072 (7)	0.0024 (6)	−0.0051 (7)
C19	0.0394 (8)	0.0564 (9)	0.0364 (7)	−0.0036 (7)	0.0023 (6)	0.0012 (7)
C20	0.0411 (8)	0.0540 (9)	0.0436 (8)	−0.0003 (7)	0.0021 (6)	0.0062 (7)
C21	0.0443 (8)	0.0447 (8)	0.0348 (7)	−0.0038 (6)	0.0048 (6)	0.0052 (6)
C22	0.0586 (10)	0.0550 (10)	0.0563 (10)	−0.0035 (8)	0.0110 (8)	−0.0088 (8)
C23	0.0531 (10)	0.0690 (12)	0.0589 (10)	−0.0227 (9)	−0.0024 (8)	0.0007 (9)
C24	0.0437 (8)	0.0598 (10)	0.0420 (8)	−0.0050 (7)	0.0037 (6)	0.0096 (7)
C25	0.0470 (8)	0.0472 (8)	0.0356 (7)	−0.0027 (7)	0.0021 (6)	0.0007 (6)
C26	0.0456 (10)	0.0882 (15)	0.0758 (13)	0.0045 (10)	0.0078 (9)	0.0121 (11)

### Geometric parameters (Å, º)

**Table:**

Cl1—C12	1.7465 (17)	C12—C13	1.378 (3)
N1—C2	1.343 (2)	C13—H13	0.9500
N1—C8	1.340 (2)	C13—C14	1.388 (2)
N2—C17	1.4597 (18)	C14—H14	0.9500
N2—C18	1.4688 (18)	C15—C16	1.510 (2)
N2—C20	1.465 (2)	C15—C19	1.508 (2)
N3—C22	1.334 (2)	C16—H16A	0.9900
N3—C23	1.330 (3)	C16—H16B	0.9900
C1—C2	1.4973 (19)	C16—C17	1.520 (2)
C1—C7	1.499 (2)	C17—H17A	0.9900
C1—C15	1.341 (2)	C17—H17B	0.9900
C2—C3	1.397 (2)	C18—H18A	0.9900
C3—C4	1.505 (2)	C18—H18B	0.9900
C3—C10	1.382 (2)	C18—C19	1.520 (2)
C4—H4A	0.9900	C19—H19A	0.9900
C4—H4B	0.9900	C19—H19B	0.9900
C4—C5	1.527 (2)	C20—H20A	0.9900
C5—H5A	0.9900	C20—H20B	0.9900
C5—H5B	0.9900	C20—C21	1.504 (2)
C5—C6	1.514 (2)	C21—C22	1.381 (2)
C6—C7	1.411 (2)	C21—C25	1.385 (2)
C6—C11	1.398 (2)	C22—H22	0.9500
C7—C14	1.399 (2)	C23—H23	0.9500
C8—H8	0.9500	C23—C24	1.391 (3)
C8—C9	1.375 (3)	C24—C25	1.385 (2)
C9—H9	0.9500	C24—C26	1.503 (2)
C9—C10	1.380 (3)	C25—H25	0.9500
C10—H10	0.9500	C26—H26A	0.9800
C11—H11	0.9500	C26—H26B	0.9800
C11—C12	1.376 (3)	C26—H26C	0.9800
			
C8—N1—C2	116.94 (15)	C1—C15—C19	123.97 (13)
C17—N2—C18	109.52 (11)	C19—C15—C16	111.07 (12)
C17—N2—C20	108.51 (12)	C15—C16—H16A	109.5
C20—N2—C18	110.72 (12)	C15—C16—H16B	109.5
C23—N3—C22	116.29 (16)	C15—C16—C17	110.72 (13)
C2—C1—C7	115.02 (12)	H16A—C16—H16B	108.1
C15—C1—C2	121.55 (13)	C17—C16—H16A	109.5
C15—C1—C7	123.42 (13)	C17—C16—H16B	109.5
N1—C2—C1	117.81 (13)	N2—C17—C16	112.43 (13)
N1—C2—C3	123.49 (14)	N2—C17—H17A	109.1
C3—C2—C1	118.70 (14)	N2—C17—H17B	109.1
C2—C3—C4	119.01 (14)	C16—C17—H17A	109.1
C10—C3—C2	117.46 (15)	C16—C17—H17B	109.1
C10—C3—C4	123.53 (15)	H17A—C17—H17B	107.9
C3—C4—H4A	109.1	N2—C18—H18A	109.3
C3—C4—H4B	109.1	N2—C18—H18B	109.3
C3—C4—C5	112.33 (13)	N2—C18—C19	111.77 (13)
H4A—C4—H4B	107.9	H18A—C18—H18B	107.9
C5—C4—H4A	109.1	C19—C18—H18A	109.3
C5—C4—H4B	109.1	C19—C18—H18B	109.3
C4—C5—H5A	107.7	C15—C19—C18	110.78 (13)
C4—C5—H5B	107.7	C15—C19—H19A	109.5
H5A—C5—H5B	107.1	C15—C19—H19B	109.5
C6—C5—C4	118.39 (13)	C18—C19—H19A	109.5
C6—C5—H5A	107.7	C18—C19—H19B	109.5
C6—C5—H5B	107.7	H19A—C19—H19B	108.1
C7—C6—C5	126.49 (14)	N2—C20—H20A	108.9
C11—C6—C5	115.21 (14)	N2—C20—H20B	108.9
C11—C6—C7	118.30 (15)	N2—C20—C21	113.25 (12)
C6—C7—C1	123.78 (14)	H20A—C20—H20B	107.7
C14—C7—C1	117.58 (13)	C21—C20—H20A	108.9
C14—C7—C6	118.61 (14)	C21—C20—H20B	108.9
N1—C8—H8	118.1	C22—C21—C20	120.91 (15)
N1—C8—C9	123.86 (17)	C22—C21—C25	117.57 (15)
C9—C8—H8	118.1	C25—C21—C20	121.50 (14)
C8—C9—H9	120.9	N3—C22—C21	124.46 (17)
C8—C9—C10	118.27 (16)	N3—C22—H22	117.8
C10—C9—H9	120.9	C21—C22—H22	117.8
C3—C10—H10	120.0	N3—C23—H23	117.6
C9—C10—C3	119.98 (17)	N3—C23—C24	124.82 (16)
C9—C10—H10	120.0	C24—C23—H23	117.6
C6—C11—H11	119.4	C23—C24—C26	121.46 (17)
C12—C11—C6	121.26 (16)	C25—C24—C23	116.89 (16)
C12—C11—H11	119.4	C25—C24—C26	121.63 (17)
C11—C12—Cl1	119.57 (14)	C21—C25—C24	119.96 (15)
C11—C12—C13	121.51 (15)	C21—C25—H25	120.0
C13—C12—Cl1	118.91 (15)	C24—C25—H25	120.0
C12—C13—H13	121.1	C24—C26—H26A	109.5
C12—C13—C14	117.74 (17)	C24—C26—H26B	109.5
C14—C13—H13	121.1	C24—C26—H26C	109.5
C7—C14—H14	118.8	H26A—C26—H26B	109.5
C13—C14—C7	122.50 (16)	H26A—C26—H26C	109.5
C13—C14—H14	118.8	H26B—C26—H26C	109.5
C1—C15—C16	124.75 (14)		
			
Cl1—C12—C13—C14	177.98 (13)	C7—C1—C15—C16	5.0 (2)
N1—C2—C3—C4	−179.86 (14)	C7—C1—C15—C19	179.20 (14)
N1—C2—C3—C10	−0.6 (2)	C7—C6—C11—C12	0.7 (2)
N1—C8—C9—C10	−0.4 (3)	C8—N1—C2—C1	−179.61 (14)
N2—C18—C19—C15	−56.69 (17)	C8—N1—C2—C3	0.0 (2)
N2—C20—C21—C22	−109.94 (17)	C8—C9—C10—C3	−0.2 (3)
N2—C20—C21—C25	71.12 (18)	C10—C3—C4—C5	−106.83 (19)
N3—C23—C24—C25	−0.3 (3)	C11—C6—C7—C1	179.20 (13)
N3—C23—C24—C26	−178.44 (19)	C11—C6—C7—C14	−2.9 (2)
C1—C2—C3—C4	−0.3 (2)	C11—C12—C13—C14	−1.7 (3)
C1—C2—C3—C10	179.00 (14)	C12—C13—C14—C7	−0.6 (2)
C1—C7—C14—C13	−179.02 (14)	C15—C1—C2—N1	−68.7 (2)
C1—C15—C16—C17	122.92 (17)	C15—C1—C2—C3	111.71 (17)
C1—C15—C19—C18	−122.30 (16)	C15—C1—C7—C6	−126.93 (17)
C2—N1—C8—C9	0.5 (3)	C15—C1—C7—C14	55.1 (2)
C2—C1—C7—C6	52.68 (19)	C15—C16—C17—N2	55.82 (17)
C2—C1—C7—C14	−125.27 (15)	C16—C15—C19—C18	52.61 (17)
C2—C1—C15—C16	−174.60 (14)	C17—N2—C18—C19	59.11 (16)
C2—C1—C15—C19	−0.4 (2)	C17—N2—C20—C21	−172.79 (13)
C2—C3—C4—C5	72.40 (19)	C18—N2—C17—C16	−58.83 (17)
C2—C3—C10—C9	0.7 (3)	C18—N2—C20—C21	66.99 (16)
C3—C4—C5—C6	−61.48 (19)	C19—C15—C16—C17	−51.94 (18)
C4—C3—C10—C9	179.93 (17)	C20—N2—C17—C16	−179.79 (13)
C4—C5—C6—C7	5.9 (2)	C20—N2—C18—C19	178.72 (12)
C4—C5—C6—C11	−173.99 (14)	C20—C21—C22—N3	−178.84 (17)
C5—C6—C7—C1	−0.7 (2)	C20—C21—C25—C24	178.02 (14)
C5—C6—C7—C14	177.28 (14)	C22—N3—C23—C24	−0.5 (3)
C5—C6—C11—C12	−179.44 (14)	C22—C21—C25—C24	−1.0 (2)
C6—C7—C14—C13	2.9 (2)	C23—N3—C22—C21	0.6 (3)
C6—C11—C12—Cl1	−178.00 (12)	C23—C24—C25—C21	1.0 (2)
C6—C11—C12—C13	1.6 (3)	C25—C21—C22—N3	0.1 (3)
C7—C1—C2—N1	111.69 (16)	C26—C24—C25—C21	179.15 (15)
C7—C1—C2—C3	−67.92 (18)		

### Hydrogen-bond geometry (Å, º)

**Table:**

*D*—H···*A*	*D*—H	H···*A*	*D*···*A*	*D*—H···*A*
C19—H19*B*···N1	0.99	2.60	3.229 (2)	121
